# Apoptotic Cells Contribute to Melanoma Progression and This Effect is Partially Mediated by the Platelet-Activating Factor Receptor

**DOI:** 10.1155/2012/610371

**Published:** 2012-04-22

**Authors:** André Luis Lacerda Bachi, Lívia Caires dos Santos, Suely Nonogaki, Sônia Jancar, Miriam Galvonas Jasiulionis

**Affiliations:** ^1^Microbiology, Immunology, and Parasitology Department, Federal University of São Paulo, 04023-900 São Paulo, SP, Brazil; ^2^Pathology Division, Adolfo Lutz Institute, 01246-000 São Paulo, Brazil; ^3^Immunology Department, University of São Paulo, 05508-900 São Paulo, SP, Brazil; ^4^Pharmacology Department, Federal University of São Paulo, 04023-032 São Paulo, SP, Brazil

## Abstract

There is evidence that the platelet-activating factor receptor (PAFR) is involved in the clearance of apoptotic cells by macrophages, and that this is associated with anti-inflammatory phenotype. Our group has previously shown that coinjection of a large number of apoptotic cells can promote tumor growth from a subtumorigenic dose of melanoma cells. Here, we studied the involvement of the PAFR in the tumor growth promoting effect of apoptotic cells. A sub-tumorigenic dose of melanoma cells (Tm1) was coinjected with apoptotic Tm1 cells, subcutaneously in the flank of C57Bl/6 mice, and the volume was monitored for 30 days. Animals received the PAFR antagonists, WEB2170 or PCA4248 (5 mg/kg body weight) or vehicle, by peritumoral daily injection for 5 days. Results showed that PAFR antagonists significantly inhibited the tumor growth induced by the coinjection of a sub-tumorigenic dose of melanoma cells together with apoptotic cells. This was accompanied by inhibition of early neutrophil and macrophage infiltration. Addition of (platelet-activating factor) to this system has no significant effect. PAFR antagonists did not affect the promoting effect of carrageenan. We suggest that the recognition of apoptotic cells by phagocytes leads to activation of PAFR pathways, resulting in a microenvironment response favorable to melanoma growth.

## 1. Introduction

Several studies have shown the importance of tumor microenvironment during several phases of tumor progression [1, 2]. The presence of inflammatory infiltrate in the tumor microenvironment, consisting mainly of macrophages, neutrophils, and lymphocytes, contributes to tumor growth by production and secretion of growth factors, metaloproteases, proangiogenic factors, cytokines, and chemokines [3].

Platelet-activating factor (PAF) is a potent phospholipid mediator which plays a major role as a primary and a secondary messenger involved in cell-to-cell communication [4]. The PAF receptor (PAFR) is a G-protein-coupled receptor present in the plasma and nuclear membrane of macrophages and other cell types [5]. Aside the key role of macrophages in innate and adaptive immunity, they are also responsible for the clearance of dying cells. This is achieved through a group of *scavenger* receptors present in the plasma membrane of macrophages with CD36 being among the best studied. More recently, it was shown that PAFR is also involved in the phagocytic removal of dying cells. De Oliveira et al. [6] showed that phagocytosis of apoptotic cells is increased compared to viable cells, and that this is reversed by a PAFR antagonist. Phagocytosis of apoptotic cells by macrophages is known to induce their polarization towards a suppressive phenotype, with the production of anti-inflammatory cytokines and mediators [7]. This is particularly relevant in the case of tumors where macrophages polarization by apoptotic cells would create an immunosuppressive environment favoring tumor growth. It was reported that murine melanoma growth was reduced by treatment with a PAFR antagonist, and when combined with chemotherapy, it increased survival time [8]. Taken together, these results suggest that during tumor growth, clearance of apoptotic cells suppresses tumor macrophages which favor tumor growth, and that this involves PAFR expressed in the macrophages plasma membrane.

Correa et al. [9] clearly demonstrated that apoptotic cells injected together with a sub-tumorigenic dose of melanoma cells promote tumor growth. Here we investigate whether this effect due to apoptotic cells is dependent on PAFR.

## 2. Material and Methods

### 2.1. Cell Culture, Reagents, and Proliferation Assay

The murine melanoma cell line Tm1 [10] was cultured in RPMI pH 6.9 (Gibco, Carlsbad, CA) supplemented with 5% fetal bovine serum (Gibco, Carlsbad, CA) at 37°C in a humidified atmosphere of 5% CO_2_ and 95% air. Carrageenan was purchased from Sigma (St. Louis, MO), WEB2170 from Boehringer Ingelheim (Germany), PCA4248 from Tocris Bioscience (USA), and PAF from Cayman Chemical (Ann Arbor, MI).

### 2.2. Tumorigenicity Assays

As previously described by Bachi et al. [11], 2 × 10^7^ Tm1 cells/mL were submitted to *γ*-irradiation (200 Gy) and to induced apoptosis, using Gammacell 300 Elan (Nordion International, Inc., Ontario, Canada), and a concentration of 2 × 10^4^ viable Tm1 cells/mL was separated. After the irradiation procedure, both cell suspensions were centrifuged and suspended in PBS, and then 50 *μ*L of each solution (1 × 10^3^ viable cells plus 1 × 10^6^ apoptotic cells) were mixed and co-injected subcutaneously into the flank of syngeneic 6- to 8-week-old C57Bl/6 female mice. To assess the effect of carrageenan on tumor progression, animals were co-injected with a mixture of 10^3^ viable Tm1 cells (50 *μ*L) and 0.1% carrageenan. Animals were kept under 12-hour daylight cycles, without food restriction and checked daily for tumor development. Each experimental group consisted of at least five animals. Tumor growth was monitored three times per week, and tumor volume was determined as follows: [(maximum diameter) × (minimum diameter)^2^] /2 [12]. Mice with a subcutaneous mass larger than 20 mm^3^ were considered positive for the presence of tumors. 

### 2.3. Immunohistochemistry Analysis

Mice were sacrificed according to institutional guidelines, and subcutaneous tissue corresponding to the site of co-injection was excised at different time points after injection (6 h, 24 h and 72 h). Hematoxylin-stained sections were used in the immunohistochemistry analysis for detection of neutrophils (myeloperoxidase-specific antibody, Santa Cruz, CA) and macrophages (F4/80-specific antibody, Abcam, Cambridge, MA). Sections were visualized on a Zeiss Axioskop 40 microscope; images were captured using a Sony Cybershot camera (3.3 megapixels) and were analyzed on a MetaVue Imaging System v.6.1 (Molecular Devices Corporation, Sunnyvale, CA) for cell counts.

### 2.4. *In Vivo *Treatment with PAF or PAF Receptor Antagonists

Platelet-activating factor (PAF) was used in different concentrations (1 *μ*g, 10 *μ*g, and 100 *μ*g), diluted in sterile PBS and injected with a sub-tumorigenic dose of Tm1 melanoma cells (10^3^ cells). Animals injected with *γ*-irradiated Tm1 cells (10^6^ cells), or carrageenan and sub-tumorigenic dose of Tm1 melanoma cells (10^3^ cells), were treated daily with 10 *μ*g of PAF in five doses (100 *μ*L), in the same site as tumor injection (termed peri-tumoral), the first dose was given together with tumor cells. The PAF receptor antagonists WEB2170 and PCA4248 were used in a concentration of 5 mg/kg of weight for each mouse, and the treatment program consisted of five peri-tumoral daily injections (100 *μ*L), the first given together with the tumor cells. All experiments were performed using groups of between five and ten, six- to-eight-week old female mice. Control groups received either injections of PBS or a sub-tumorigenic dose of Tm1 melanoma cells (10^3^ cells), according to the type of experiment (see Section 3 and figure legends).

### 2.5. Statistical Analysis

The data are expressed as the mean ± SD. Nonpaired nonparametric Student's *t*-test was used to analyze differences between the means, using the InStat software package. The significance level was established at *P* < 0.05.

## 3. Results

**Figure 1:**
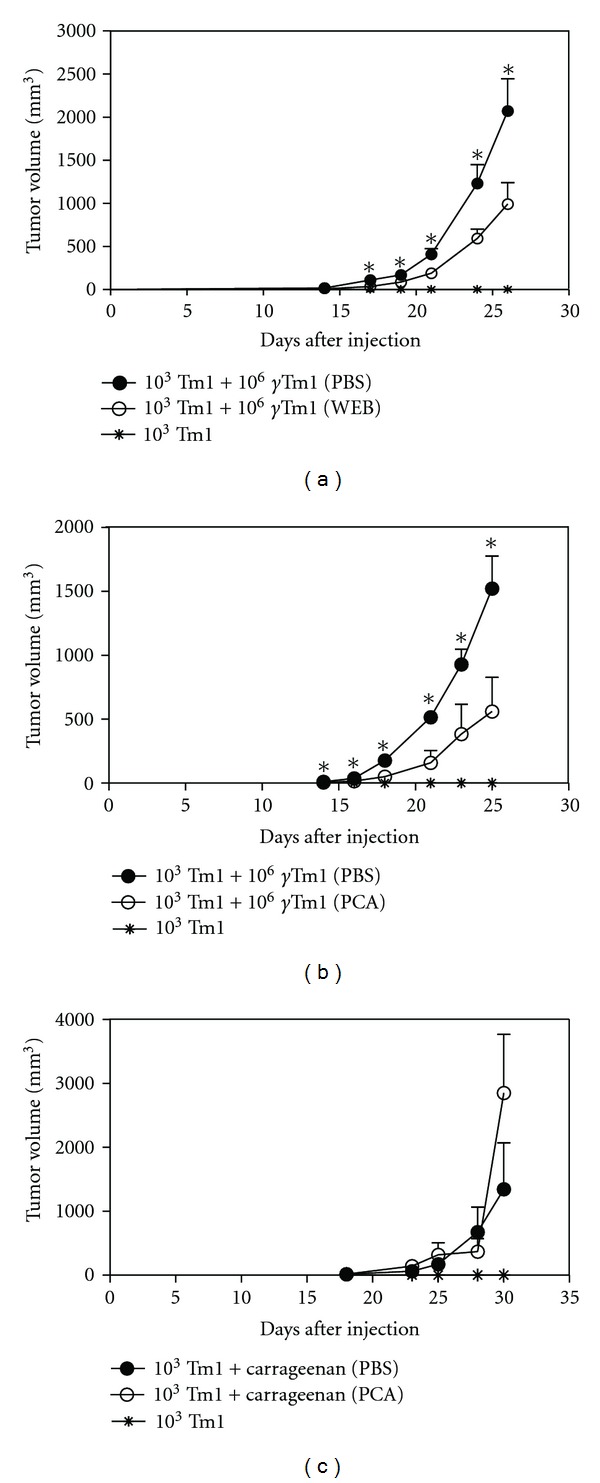
PAFR antagonists reduce melanoma growth associated with a massive number of apoptotic cells. The treatment of C57Bl/6 mice with PAFR antagonists showed a significant reduction in the melanoma growth (10^3^ Tm1 viable cells) induced by apoptotic cells (10^6^ *γ*-irradiated Tm1 cells, (a) and (b)) but not by carrageenan (0.1% (c)). The animals were treated with PBS or PAFR antagonists (WEB2170 (WEB, (a)) or PCA4248 (PCA, (b) and (c)) 5 mg/kg body weight)) by peritumor daily injections during 5 days. All experiments were performed using groups of 5 to 10 mice, which were considered harboring tumors when the subcutaneous mass reached 20 mm^3^ (palpable tumor). *Denotes statistical significance (*P* < 0.05) using nonpaired nonparametric Student's *t*-tests.

In a previous work we have established a protocol where the co-injection of a sub-tumorigenic dose of melanoma cells (10^3^), together with apoptotic cells (10^6^), or carrageenan, promoted melanoma growth [11]. Here we used the same model to investigate the involvement of PAFR. Animal groups submitted to either co-injection (10^6^ *γ*-irradiated tumor cells and 10^3^ viable tumor cells) or injection of carrageenan and 10^3^ viable tumor cells were treated with the selective PAFR antagonists WEB2170 or PCA4248.  Figure 1 shows that treatment with WEB2170 (Figure 1(a)) or PCA4248 (Figure 1(b)) reduced tumor progression, suggesting that the PAFR plays an important role in growth of a sub-tumorigenic dose of melanoma cells induced by a large number of apoptotic cells. Injection of the classic inflammatory agent, carrageenan, also promoted the growth of a sub-tumorigenic dose of melanoma cells, as previously reported; however, the treatment with PAFR antagonists did not affect tumor growth (Figure 1(c)). These results suggest that PAFR contributes to the growth of a sub-tumorigenic dose of tumor cells associated with co-injection of apoptotic cells. 

 To evaluate if PAF alone was able to promote the growth of a sub-tumorigenic dose of melanoma cells, three different doses of PAF (1 *μ*g, 10 *μ*g, and 100 *μ*g) were injected together with 10^3^ viable tumor cells. We could not observe tumor growth even up to sixty days following the injection (data not shown). 

**Figure 2:**
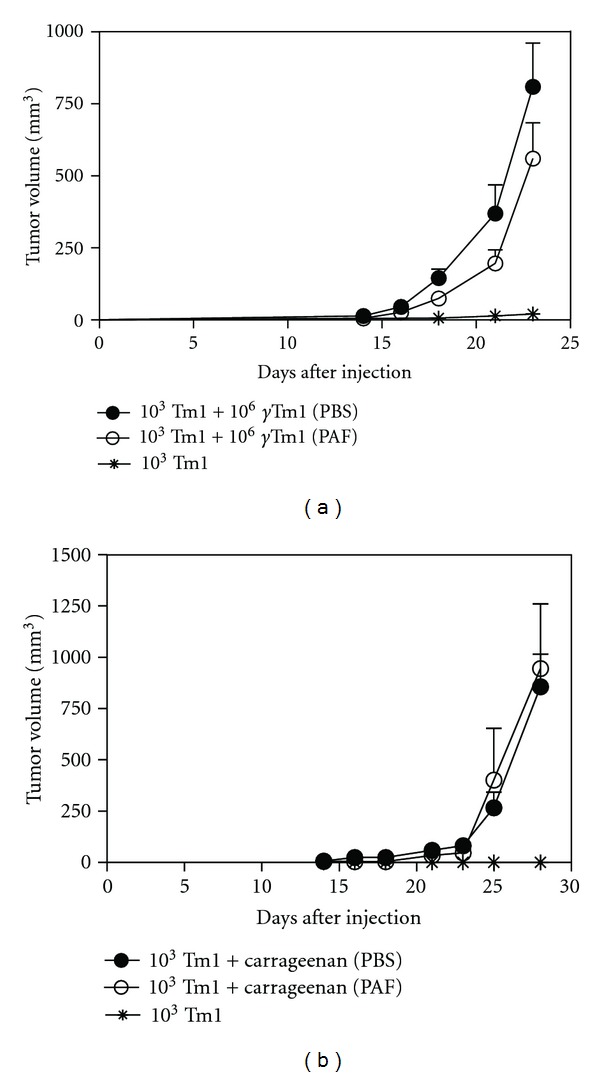
PAF does not affect melanoma progression induced by apoptotic cells or carrageenan. Melanoma growth (10^3^ viable Tm1 cells) induced by apoptotic cells (10^6^ *γ*-irradiated Tm1 cells, (a)) or carrageenan (0.1%, (b)) in C57Bl/6 mice did not show any statistical difference between the treatment with PBS or PAF (10 *μ*g) given by peritumor injection daily for 5 consecutive days.

 Next, we evaluated if injection of PAF would modify the tumor growth induced by co-injection of apoptotic cells or carrageenan. Animals co-injected with apoptotic cells (Figure 2(a)), or with carrageenan and 10^3^ viable tumor cells (Figure 2(b)), were treated for five days with 10 *μ*g of PAF. No significant statistical difference was observed in the tumor growth in these conditions.

**Figure 3:**
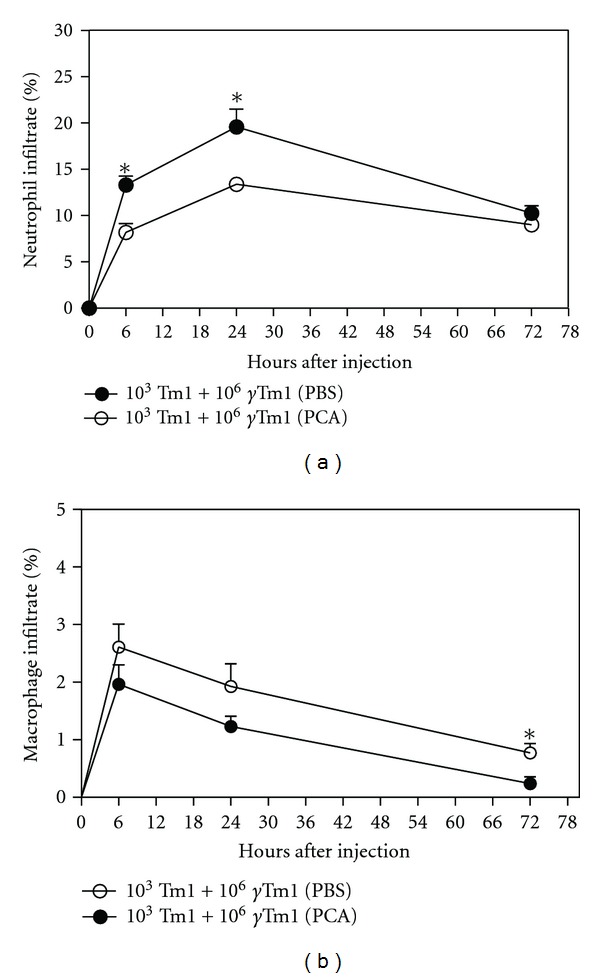
Treatment with PAFR antagonist reduces neutrophil and macrophage infiltration. The inhibition of PAFR using PCA4248 (PCA) results in reduced neutrophil and macrophage infiltration in mice submitted to coinjection of apoptotic cells (10^6^ *γ*-irradiated Tm1) and a subtumorigenic dose of Tm1 melanoma cells (10^3^ viable cells). Subcutaneous tissues from animals treated with PBS or PCA4248 (PCA), for 5 consecutive days, were surgically removed 6, 24 and 72 h after coinjection and processed for immunohistochemistry analysis using specific antibodies against myeloperoxidase (antigen expressed mainly by neutrophils, (a)) and F4/80 (cell marker by macrophages, (b)). *Denotes statistical significance (*P* < 0.05) using nonpaired nonparametric Student's *t*-tests.

Subcutaneous tissues from animals co-injected with apoptotic cells (10^6^) and viable melanoma cells (10^3^) and treated with PAF receptor antagonist PCA4248 or PBS (control) were surgically removed after 6 h, 24 h, and 72 h of the co-injection to evaluate the infiltration of macrophages and neutrophils by immunohistochemical analysis for MPO expression, as marker of neutrophils and F4/80, as marker of macrophages. Analyses showed a significant reduction in the expression of myeloperoxidase (MPO), 6 h and 24 h after co-injection in the group of animals that were treated with PCA4248. Seventy-two hours after co-injection, the expression of MPO decreased and was similar in both groups of animals (Figure 3(a)). Regarding macrophage infiltration, in contrast to neutrophils, the number of cells expressing F4/80 did not differ between the groups at 6 h and 24 h after co-injection. However, after 72 h of co-injection the analysis showed a significant reduction of F4/80 expression in animals treated with PCA4248 in comparison to the group of animals treated with PBS (Figure 3(b)).

## 4. Discussion

 According to our previous data, the injection of a large number of apoptotic cells creates a tumor microenvironment that favors the growth of a sub-tumorigenic dose of melanoma cells [9, 11]. During the process of apoptosis, different lipids, particularly phospholipids, which are exposed at the cell membrane are oxidized. Oxidised moieties present in apoptotic cells may be recognized by scavenger receptors, such as CD36, expressed in phagocytes, and may induce the clearance of dying cells. The PAFR is also involved since it has been shown that PAFR antagonists inhibit the phagocytosis of apoptotic and necrotic cells by macrophages *in vitro* [6]. Here we showed that the specific inhibition of PAFR by the antagonist WEB2170 reduced significantly the growth of a sub-tumorigenic dose of melanoma cells associated with a large number of apoptotic cells. Confirming the role of this receptor in promoting tumor growth, the inhibition of PAFR by a second antagonist (PCA4248) partially abrogated the tumor growth associated with the apoptotic cells. The two antagonists used in this study both reduced significantly the tumor growth, but PCA4248 was more effective than WEB2170. Although the two antagonists are more or less equipotent at a molar ratio, the half time of duration of action could be different for each compound. These data suggest that interaction of PAF-like molecules on the surface of apoptotic cells with PAFR in cells present in the tumor microenvironment exerts an important modulation during the initial stages following tumor cell implantation.

We also observed that co-injection of PAF with a sub-tumorigenic dose of melanoma cells does not have the growth promoting effect of the apoptotic cells (data not shown). Moreover, addition of PAF to the co-injection model did not affect the tumor growth. Although PAF is an unstable compound, the high dose that was injected (10 *μ*g) in the site of the tumor for five consecutive days should be effective. Fernandez-Gallardo et al. [13] reported that even ten-times less PAF, injected into mouse skin, was able to induce plasma extravasation.

These results show that activation of the PAFR by exogenous PAF does not reproduce the effect of the apoptotic cells and suggest that the recognition of other moieties, besides PAF-like ones, present on apoptotic cells is required. It has been shown by Rios et al. [14] that for optimal uptake of oxLDL by human monocytes/macrophages both the scavenger receptor CD36 and PAFR are required. In subsequent work, these authors also demonstrated that following phagocytosis of apoptotic cells, PAFR colocalizes with CD36 in the plasma membrane of macrophages. Moreover, in HEK293 cells transfected with PAFR, CD36, or both, apoptotic cells only induce IL-8 production when both receptors are present [15]. This allows the speculation that engagement of *scavenger* receptors by apoptotic cells recruits PAFR to specific membrane microdomains, and that the signaling elicited by each receptor converges to activate gene transcription with the results being production of cytokines which modify the microenvironment.

 The microenvironment surrounding the tumor mass will contain proliferating tumor cells, many of which will be undergoing apoptosis and necrosis, along with several host components that include stromal cells, and a characteristic inflammatory infiltrate associated with constant tissue remodeling [16]. Stromal cells, which are mainly comprised of fibroblasts, macrophages, neutrophils, eosinophils, mast cells, lymphocytes, dendritic cells and endothelial cells, form a part of the tumor microenvironment which supports and regulates the growth of tumor cells [17]. Interactions between tumor and stromal cells can occur via cell-cell interactions, or by cytokine- or chemokine-mediated signaling [18]. We propose here that these interactions also involve PAFR-mediated signaling. Immunochemistry assays on tumors from mice injected with apoptotic cells and a sub-tumorigenic dose of melanoma cells showed that treatment with the PAFR antagonist reduced neutrophil infiltration in the first 24 h of tumor injection and after three days, infiltration of macrophages. It can be speculated that the inhibition of macrophage infiltration by the PAFR antagonist at the early phase of tumor/apoptotic cell co-injection would reduce the level of suppressor molecules in the tumor microenvironment favoring the inhibition of tumor progression observed with PAFR antagonist treatment. Regarding neutrophils, more studies are needed to understand their role in tumor growth.

 During tumor development a large number of tumor cells become apoptotic or necrotic. These dead cells are removed through phagocytosis by neighboring and inflammatory cells, mainly macrophages. It was demonstrated that phagocytosis of apoptotic cells reduces macrophage activation [7]. As a consequence, the presence of apoptotic cells can favor tumor growth by suppressing macrophage functions. The PAFR has been shown to participate in the phagocytic removal of apoptotic cells by macrophages, and this is followed by production of suppressor molecules [6]. It has been also shown that PAFR antagonists inhibit melanoma growth [8]. Here we demonstrate that apoptotic cells can promote tumor growth through PAFR-dependent mechanisms.
